# Spatial clustering of domestic violence attitudes toward women in Ghana

**DOI:** 10.1371/journal.pgph.0003261

**Published:** 2024-05-28

**Authors:** Cecilia Richardsen, Djibril M. Ba, Anna E. Ssentongo, Paddy Ssentongo

**Affiliations:** 1 Penn State College of Medicine, Hershey, Pennsylvania, United States of America; 2 Department of Public Health Sciences, Penn State College of Medicine, Hershey, Pennsylvania, United States of America; 3 Department of Medicine, Penn State Milton S Hershey Medical Center, Hershey, Pennsylvania, United States of America; University College London, UNITED KINGDOM

## Abstract

Violence against women is a global public health issue associated with increased morbidity and mortality. The United Nations defines violence against women as “any act of gender-based violence that results in, or is likely to result in, physical, sexual, or mental harm or suffering to women, including threats of such acts, coercion or arbitrary deprivation of liberty, whether occurring in public or in private life”. There is paucity of data on the spatial distribution and predictors of violence against women in sub-Saharan Africa. The objective of this study was to investigate the geographical distribution of attitudes toward wife beating in Ghana, a sub-Saharan African country, utilizing data from the 2014 Ghana Demographic and Health Survey (DHS). Participants from over eleven thousand households were surveyed on topics of demographics and justification of wife beating in at least one of five different scenarios. The identification of geographic clusters of men and women who endorsed wife beating was performed using Ripley K functions. The comparison of the spatial distributions of women and men justifying wife beating were performed using spatial relative risk surfaces. The spatial analysis indicated the presence of clusters in women and men’s approval for wife beating compared to those who do not approve of wife beating, with a statistical significance level set at p < 0.01. Major spatial clusters of approval of wife beating were in the Northern region, for both men and women, and in the Upper West region of Ghana for the men participants. This is the first study to explore the geographical distribution of attitudes toward wife beating in Ghana, and revealed evidence of several regional heterogeneous clusters where wife beating was more commonly justified by both men and women. Targeted intervention for reducing the justification of wife beating in Ghana should be focused in these regions.

## Introduction

According to a report from the World Health Organization in 2018, at least 736 million, or 1 in 3, women aged 15 or older have experienced intimate partner violence or non-partner sexual violence in their lifetime [1]. They define violence against women as “any act of gender-based violence that results in, or is likely to result in, physical, sexual, or mental harm or suffering to women, including threats of such acts, coercion or arbitrary deprivation of liberty, whether occurring in public or in private life” [1]. Violence against women is associated with increased morbidity and mortality. A previous study investigating health outcomes and domestic violence found that exposure to domestic violence was associated with increased prevalence of depression, sleep problems, abortion, pain, and hypertension [2]. This type of abuse also has implications for children who are exposed to domestic violence, as they are at increased risk of becoming victims of abuse and developing emotional and behavioral problems [3].

The distribution of domestic violence is not uniform across the globe. Risk factors include low level of gender equality, low levels of women’s access to paid employment, and harmful masculine behaviors [1]. There are several studies that have investigated domestic violence in Ghana. One considered the determinants of domestic violence against women in Ghana and found that place of residence, alcohol use by husband, and family history of violence increased the risk that a woman would experience domestic violence [4]. This study utilized data from the 2008 Ghana Demographic and Health Surveys (DHS) [5], which may now be outdated. A second study considered the endorsement of wife beating in Ghana using more recent data from the 2014 Ghana DHS and found associations between several factors and both men and women’s approval of wife beating [6]. To date, there is no study estimating the spatial distribution or clustering of attitudes toward wife beating in Ghana.

The purpose of this study is to fill the gap of knowledge about the geographical distribution of attitudes toward wife beating in Ghana. This knowledge may provide opportunities for targeted intervention in specific locations in Ghana to change attitudes toward wife beating, and furthermore to decrease the prevalence of violence against women. This is the first spatial mapping of attitudes toward wife beating in Ghana using data from the 2014 DHS in Ghana.

## Materials and methods

### Study population

The data for the study were obtained from the 2014 Ghana DHS [7]. The survey was implemented by the Ghana Statistical Service, the Ghana Health Service, and the National Public Health Reference Laboratory. This nationally representative survey included interviews from 11,835 households, comprised of 9,396 women and 4,388 men who were recruited for the surveys according to DHS protocol. The women were between the ages of 15 and 49 years, and the men were between the ages of 15 and 59 years. Participants were surveyed on topics of type of residence, highest education level, religion, literacy, wealth, health insurance status, marital status, employment status, and HIV testing status. Individual participants were not able to be identified by the authors at any point during the study due to geomasking procedures performed by the DHS, which is a standard process that is used to protect the confidentiality of the participants. The DHS is designed to provide a representative sample for data analysis. The data report was used to identify the demographic information and primary variable outcomes.

### Primary variables

The primary outcome was the justification of wife beating in at least one of five different scenarios. The five scenarios include if the wife goes out without telling the husband, if the wife neglects the children, if the wife argues with the husband, if the wife refuses to have sex with the husband, and if the wife burns the food. Participants answered “yes” or “no” or “don’t know” to these questions.

### Statistical analysis

Sociodemographic information and prevalence of approval of wife beating were reported using descriptive analysis of frequencies. No confidence intervals were generated for these. Using the binary variables for the primary outcome—justification of wife beating in at least one of five different scenarios—we identified geographic clusters of men and women who endorsed wife beating was performed using Ripley’ K functions [8]. The K-function is a tool to evaluate completely mapped spatial point process data. The assumption is that the spatial distribution of the points or feature have a complete spatial randomness (CSR). When the observed K value is larger than the expected K value for a particular distance, the distribution is more clustered than a random distribution at that distance. When the observed K value is smaller than the expected K value, the distribution is more dispersed than a random distribution at that distance. We constructed the 95% confidence estimate of random clustering from Monte Carlo permutation of points in 500 trials.

Comparison of the spatial distributions of women and men justifying wife beating were performed using spatial relative risk surfaces [9]. Statistical significance level was set at p < 0.01 for Ripley’s K analysis. All analyses were performed with the R statistical software [10]. R script and code to reproduce the data: https://github.com/ssentongojeddy/Violence_Women

### Ethics statement

The 2014 Ghana DHS was reviewed and approved by the ICF Institutional Review Board. ICF’s IRB complied with the United States Department of Health and Human Services requirements for the Protection of Human Subjects 45 CFR 46 in making this determination.

## Results

### Demographics

As shown in Table 1, the ages of women ranged from 15 to 49 and the ages of men ranged from 15 to 59. An estimated 49% of women and 46.7% of men resided in urban areas. Over half of respondents had their highest level of education at the secondary level, with 51.7% of women and 59% of men reaching this level. Few had completed higher levels of education. Most participants identified with a denomination of the Christian religion, and about a fifth of participants identified with Islam. There was a difference in the level of literacy between men and women, with 49% of women being unable to read at all, and 29.2% of men not being able to read at all. About a quarter of participants belonged to the lowest wealth quintile, with 24.9% women and 26.3% men being in the lowest category. At least half reported having health insurance coverage, with 66% women and 53% men. Nearly half of both women and men were married, with 45.2% of women and 44.8% of men being married. Most were currently working, with 72% women and 83.4% men. Most had not been tested for HIV, with 53.5% women and 78.4% men.

**Table 1:** Background characteristics of surveyed participants in the 2014 demographic and health survey in Ghana.

	Women N (%)	Men N (%)
	N = 9396	N = 4388
***Age***		
**15–19**	1756(18.7)	889(20.3)
**20–24**	1571(16.7)	620(14.1)
**25–29**	1564(16.7)	577(13.1)
**30–34**	1343(14.3)	497(11.3)
**35–39**	1260(13.4)	472(10.8)
**40–44**	1032(11)	442 (10.1)
**45–49**	870(9.3)	358(8.2)
**50–54**	0(0)	302 (6.9)
**55–59**	0(0)	231 (5.3)
***Type of Residence***		
**Urban**	4602(49)	2050 (46.7)
**Rural**	4794(51)	2338 (53.3)
***Highest Education Level***		
**No education**	2281(24.3)	656(14.9)
**Primary**	1747(18.6)	696(15.9)
**Secondary**	4854(51.7)	2591(59)
**Higher**	514(5.5)	445(10.1)
***Religion***		
**Catholic**	1341(14.3)	619(14.1)
**Anglican**	72(0.8)	31(0.7)
**Methodist**	547(5.8)	233(5.3)
**Presbyterian**	513(5.5)	242(5.5)
**Pentecostal/charismatic**	3457(36.8)	1128(25.7)
**Other Christian**	1239(13.2)	682(15.5)
**Islam**	1726(18.4)	926(21.1)
**Traditional/spiritualist**	226(2.4)	264(6)
**No religion**	273(2.9)	261(5.9)
**Other**	1(0)	2 (0)
***Literacy***		
**Cannot read at all**	4604(49)	1281 (29.2)
**Able to read only parts of sentence**	873(9.3)	497(11.3)
**Able to read whole sentence**	3900(41.5)	2602(59.3)
**No card with required language**	11(0.1)	3(0)
**Blind/visually impaired**	3(0)	3 (0)
***Wealth Index***		
**Lowest quintile (Poorest)**	2335(24.9)	1154(26.3)
**Poorer**	1759(18.7)	850(19.4)
**Middle**	1902(20.2)	807(18.4)
**Richer**	1771(18.9)	815(18.6)
**Highest quintile (Richest)**	1629(17.3)	762(17.4)
***Covered by health insurance***		
**No**	3196(34)	2063 (47)
**Yes**	6197(66)	2324 (53)
***Current marital status***		
**Never in union**	3041(32.4)	1866(42.5)
**Married**	4243(45.2)	1967(44.8)
**Living with partner**	1213(12.9)	335(7.6)
**Widowed**	269(2.9)	38(0.9)
**Divorced**	260(2.8)	98(2.2)
**No longer living together/separated**	370(3.9)	84(1.9)
***Currently working***		
**No**	2626(28)	727 (16.6)
**Yes**	6761(72)	3660 (83.4)
***Ever been tested for HIV***		
**No**	5021(53.5)	3438(78.4)
**Yes**	4370(46.5)	950(21.6)

### Prevalence of attitudes of justification toward wife beating

As shown in **Table 2**, overall, 31% of women and 15% of men justified wife beating in at least one of five scenarios. The prevalence of women who endorsed that wife beating was justified if the wife goes out without telling the husband was 18.3% and the prevalence men who endorsed that wife beating was justified if the wife goes out without telling the husband was 7.7%. The prevalence for both women and men who endorsed that wife beating was justified if the wife neglects the children were 23.4% and 9.8%, respectively. There were 17.6% of women and 6.9% of men who endorsed that wife beating was justified if the wife argues with the husband. There were 13.8% of women and 5.6% of men who endorsed that wife beating was justified if the wife refuses to have sex with the husband. There were 8.8% of women and 3.3% of men who endorsed that wife beating was justified if the wife burns the food. The DHS survey tracked the number of women and men who answered yes to any of these five scenarios and included it in the data report.

**Table 2:** Justification for wife beating by men and women in five different scenarios.

	Women N (%)	Men N (%)
	N = 9396	N = 4388
***Wife beating justified if wife goes out without telling husband***	N (%)	N (%)
**No**	762(81.2)	4033(91.9)
**Yes**	1721(18.3)	337(7.7)
**Don’t Know**	46(0.5)	17(0.4)
***Wife beating justified if wife neglects children***		
**No**	7151(76.1)	3940(89.8)
**Yes**	2199(23.4)	432(9.8)
**Don’t Know**	44(0.5)	15(0.3)
***Wife beating justified if wife argues with husband***		
**No**	7694(81.9)	4063(92.6)
**Yes**	1649(17.6)	304(6.9)
**Don’t Know**	50(0.5)	19(0.4)
***Wife beating justified if wife refuses to have sex with husband***		
**No**	8034(85.5)	4106(93.6)
**Yes**	1299(13.8)	247(5.6)
**Don’t Know**	61(0.6)	34(0.8)
***Wife beating justified if wife burns the food***		
**No**	8531(90.8)	4230(96.4)
**Yes**	828(8.8)	145(3.3)
**Don’t Know**	35(0.4)	12(0.3)
***Answered yes to any justification of wife beating***		
**Yes**	2911(31)	652(14.9)

### Geospatial analysis

**Fig 1:**
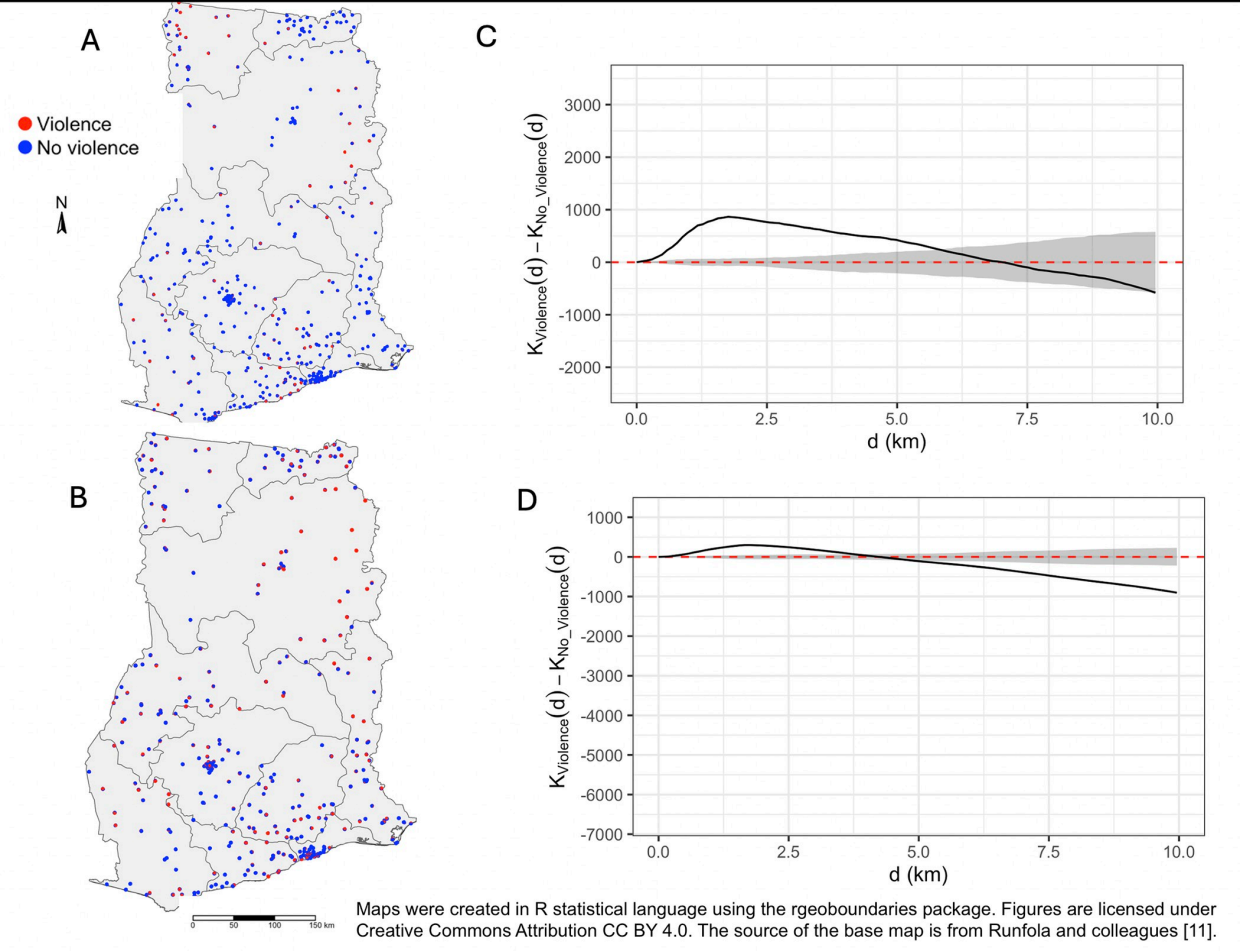
Geographic distribution of study participants who justified violence against wives (red dot) and those who did not justify violence (blue circles) in regions of Ghana. Panel (**A**) displays male participants and (**B**) female participants. Ripley’s K function for the difference in individuals who endorse and those who do not endorse wife violence (black line) shows a spatial aggregation of individuals who justify violence against wives at a 0.5 to 6 km scale, defined by the 95% confidence bounds from Monte Carlo simulations (grey) for men study participants who endorse violence against intimate partners compared to those who do not (**C**) and spatial aggregation at 1 to 4 km for the women who endorses violence against intimate partners compared to those who do not (**D**). Maps were created in R statistical language using the *rgeoboundaries* package [11].

**Fig 2:**
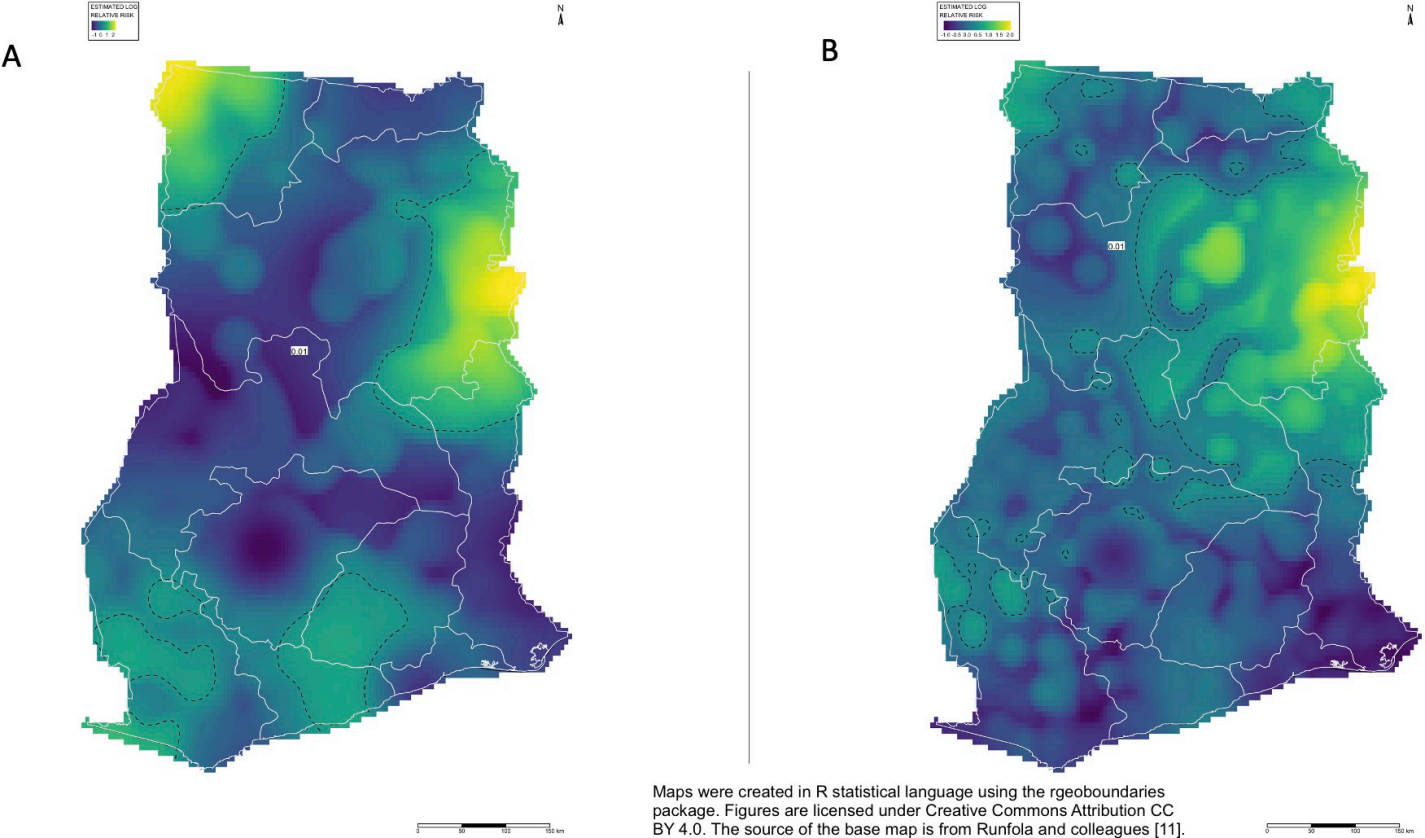
Spatial clustering of wife beating attitudes. Spatial clustering of men approving of wife beating (A) and women justifying being beaten by husband (B) in one of the five scenarios described. Maps were created in R statistical language using the *rgeoboundaries* package [11].

Displayed in Fig 1 were the geographic locations of participants stratified by their justification of wife beating. Ripley’s K spatial analysis indicated presence of clusters in women and men’s approval for wife beating compared to those who do not approve wife beating in at least one of the five scenarios described. The average size of cluster was larger for men participants than women participants. The location of spatial clustering of men who justify wife beating was largely similar to that of women who responded that wife beating was justified in at least one of the five scenarios, as displayed in Fig 2. Major spatial clusters for the justification of wife beating were in the Northern region, for both men and women. An additional major cluster of justification for wife beating in the Upper Western region of Ghana was observed among men participants. Maps were created in R statistical language using the *rgeoboundaries* package. Figures are licensed under Creative Commons Attribution CC BY 4.0. The source of the base map is from Runfola and colleagues [11].

## Discussion

### Key findings

This study investigated the prevalence of the justification of wife beating in Ghana, a sub-Saharan African country, utilizing data from a representative sample of over 13,000 individuals. This is the first study to explore the geographical distribution of attitudes toward wife beating in Ghana, and revealed evidence of several regional heterogeneous clusters where wife beating was more commonly justified by both men and women.

### Prior research

There have been several previous studies regarding domestic violence in Ghana. Studies that utilized data from the 2003 and 2008 Ghana DHS to study attitudes toward wife beating concluded that younger age, lower level of education, rural area of residence, traditional faith practice, husband’s use of alcohol, family history of violence, and lower wealth index were associated with Ghanaian women’s approval of domestic physical violence toward women [12]. A similar study utilized data from the 2014 Ghana DHS also concluded that younger age, lower level of education, and lower wealth index were associated with both Ghanaian men’s and women’s approval of wife beating [6]. While these studies have provided valuable insight to the factors associated with domestic violence in Ghana, this is the first study to perform an analysis with advanced statistical methods that shows locations with more prevalent justifications of wife beating.

### Geospatial clusters

For men’s attitudes toward wife beating, there were two primary clusters where these actions were justified, which occurred in the Upper West Region and the Northern Region, with some extension into the Oti Region. There were also four small clusters in the Eastern, Central, Western, and Western North Regions. For women’s attitudes toward wife beating, the primary cluster occurred in the Northern Region, with several small clusters throughout the northern, western, and southern areas of the country. While the determination of factors associated with the justification of wife beating in each spatial cluster was outside of the scope of this study, there are several parallels that may be considered. The 2015 Ghana Poverty Mapping Report conducted by the Ghana Statistical Service determined that the Upper West Region had the highest poverty incidence among all regions in Ghana, with the highest poverty headcount and the highest depth of poverty [13]. This is supported by prior studies that have identified an association between lower wealth index and domestic violence [4, 6, 14]. A 2020 UNICEF report on education in Ghana using multiple indicator cluster surveys showed that the Upper West, Upper East, and Northern regions had the lowest completion rates of primary education throughout the country [15]. Targeted intervention for reducing the justification of wife beating in Ghana may involve a focus on addressing poverty and education in the Upper West Region and the Northern Region, though further research is necessary. This process may also involve counseling and education of women to improve self-esteem and empowerment.

### Public health implications

The United Nations has identified gender equality as one of the seventeen goals in the 2030 Agenda for Sustainable Development. This includes minimizing violence against women, which has affected at least 736 million women over the age of 15 between 2000 and 2018 [16]. There is evidence that domestic violence has both short and long term physical and mental health consequences, including headaches, chronic stress, gastrointestinal disorders, gynecological disease, and depression [17]. Domestic violence toward women has adverse effects that extend even beyond the woman who was mistreated. A study on intimate partner violence in Africa found that adolescents who were exposed to domestic violence in the home had health implications in adulthood, including anxiety, distress, and further perpetuation of intimate partner violence [18]. Another study identified an association between lower women’s empowerment and higher rates of neonatal, infant, and under-5 mortality in low- and middle-income countries, revealing that empowering women may be one method to reduce preventable child deaths [19]. In this study, a larger percentage of women responded “yes” to scenarios where wife beating was justified than men. This may be an indication that women in Ghana included in this study do not feel empowered, as 31% of women responded that they deserve to be beaten by their husband in at least one of the five scenarios given. Domestic violence is a cause of morbidity and mortality across the globe and demands further policy development and intervention. These issues increase healthcare costs, affect the productivity of communities, and result in lasting physical and psychological trauma. It is presumed that there is a link between attitudes of approval of wife beating and the prevalence of physical violence put into action, though additional research is necessary to solidify this connection. The identification of geographical areas that have a higher prevalence of attitudes that endorse wife beating may aid in providing a more targeted approach to addressing this public health issue in low resource areas.

### Strengths and limitations

There are several strengths of this study. First, data was utilized from a national survey in Ghana, which is designed to be representative of the entire country. Second, to the best of our knowledge, this study is the first to identify geographical locations in Ghana where wife beating is justified. A limitation to consider is that the data are focused on situations where wife beating is justified, making it difficult to assess the true prevalence of domestic violence. A second limitation is related to the geographic displacement utilized by the DHS, which is a process that was designed to protect the identities of the survey respondents. While the release of precise geographic coordinates from the DHS surveys have been geomasked, great lengths have been taken to preserve the geospatial patterns of the regions surveyed [20].

### Conclusions and recommendations

Our findings suggest that there are several heterogeneous geographical clusters in Ghana where wife beating is more commonly justified by both men and women. Future studies should investigate the determining factors of the justification of wife beating that are associated with each identified spatial cluster.

## References

[1] World Health Organization. Violence against women prevalence estimates, 2018: global, regional and national prevalence estimates for intimate partner violence against women and global and regional prevalence estimates for non-partner sexual violence against women. 2018 [cited 14 April 2022]. Available from: https://www.who.int/publications/i/item/9789240026681 10.1016/S0140-6736(21)02664-7 PMC8885817 35182472

[2] Hawcroft C, Hughes R, Shaheen A, Usta J, Elkadi H, Dalton T, et al. Prevalence and health outcomes of domestic violence amongst clinical populations in Arab countries: a systematic review and meta-analysis. BMC Public Health. 2019;19: 315. doi: 10.1186/s12889-019-6619-2 30885168 PMC6421940

[3] Holt S, Buckley H, Whelan S. The impact of exposure to domestic violence on children and young people: A review of the literature. Child Abuse & Neglect. 2008;32: 797. doi: 10.1016/j.chiabu.2008.02.004 18752848

[4] Adjah ESO, Agbemafle I. Determinants of domestic violence against women in Ghana. BMC Public Health. 2016;16: 368. doi: 10.1186/s12889-016-3041-x 27139013 PMC4852424

[5] Ghana Statistical Service (GSS), Ghana Health Service (GHS), and ICF Macro. 2009. Ghana Demographic and Health Survey 2008. Accra, Ghana: GSS, GHS, and ICF Macro.

[6] Dickson KS, Ameyaw EK, Darteh EKM. Understanding the endorsement of wife beating in Ghana: evidence of the 2014 Ghana demographic and health survey. BMC Women’s Health. 2020;20: 25. doi: 10.1186/s12905-020-00897-8 32046703 PMC7011351

[7] Ghana Statistical Service (GSS), Ghana Health Service (GHS), and ICF International. 2015. Ghana Demographic and Health Survey 2014. Rockville, Maryland, USA: GSS, GHS, and ICF International.

[8] Diggle PJ, Chetwynd AG. Second-Order Analysis of Spatial Clustering for Inhomogenous Populations. Biometrics. 1991;47: 1155. doi: 10.2307/2532668 1742435

[9] Kelsall JE, Diggle PJ. Non-parametric estimation of spatial variation in relative risk. Statistics in Medicine. 1995;14: 2335. doi: 10.1002/sim.4780142106 8711273

[10] R Development Core Team 2020 version 3.0.6.

[11] Runfola D, et al. geoBoundaries: A global database of political administrative boundaries. PLoS ONE. 2020;15(4): e0231866. doi: 10.1371/journal.pone.0231866 32330167 PMC7182183

[12] Doku DT, Asante KO. Women’s approval of domestic physical violence against wives: analysis of the Ghana demographic and health survey. BMC Women’s Health. 2015;15: 120. doi: 10.1186/s12905-015-0276-0 26691763 PMC4687112

[13] Ghana Statistical Service. Ghana Poverty Mapping Report, 2015. 2015 [cited 19 March 2022].

[14] Semahegn A, Mengistie B. Domestic violence against women and associated factors in Ethiopia; systematic review. Reproductive Health. 2015;12: 78. doi: 10.1186/s12978-015-0072-1 26319026 PMC4553009

[15] UNICEF. Ghana Education Fact Sheets: Analysis for learning and equity using MICS data, 2020. 2020 [cited 27 April 2022].

[16] United Nations. Sustainable Development, 2021. 2021 [cited 25 April 2022]. Available from: https://sdgs.un.org/goals/goal5

[17] Campbell J, Jones AS, Dienemann J, Kub J, Schollenberger J, O’Campo P, et al. Intimate Partner Violence and Physical Health Consequences. Arch Intern Med. 2002;162: 1157. doi: 10.1001/archinte.162.10.1157 12020187

[18] Roman NV, Frantz JM. The prevalence of intimate partner violence in the family: a systematic review of the implications for adolescents in Africa. Family Practice. 2013;30: 256. doi: 10.1093/fampra/cms084 23363539

[19] Doku DT, Bhutta ZA, Neupane S. Associations of women’s empowerment with neonatal, infant and under-5 mortality in low- and /middle-income countries: meta-analysis of individual participant data from 59 countries. BMJ Global Health. 2020;5: 1558. doi: 10.1136/bmjgh-2019-001558 32133162 PMC7042599

[20] Burgert C, Colston J, Roy T, & Zachary B. Geographic Displacement Procedure and Georeferenced Data Release Policy for the Demographic and Health Surveys, 2013. 2013 [cited 17 September 2022]. Available from: https://dhsprogram.com/pubs/pdf/SAR7/SAR7.pdf

